# Transcriptional reprogramming caused by the geminivirus *Tomato yellow leaf curl virus* in local or systemic infections in *Nicotiana benthamiana*

**DOI:** 10.1186/s12864-019-5842-7

**Published:** 2019-07-04

**Authors:** Mengshi Wu, Xue Ding, Xing Fu, Rosa Lozano-Duran

**Affiliations:** 1 Shanghai Center for Plant Stress Biology, CAS Center for Excellence in Molecular Plant Sciences, Chinese Academy of Sciences, Shanghai, 201602 China; 2 University of the Chinese Academy of Sciences, Beijing, 100049 China

**Keywords:** Geminivirus, Transcriptome, *Nicotiana benthamiana*, TYLCV, Agroinoculation

## Abstract

**Background:**

Viruses have evolved to create a cellular environment permissive for viral replication in susceptible hosts. Possibly both enabling and resulting from these virus-triggered changes, infected hosts undergo a dramatic transcriptional reprogramming, the analysis of which can shed light on the molecular processes underlying the outcome of virus-host interactions. The study of the transcriptional changes triggered by the plant DNA viruses geminiviruses is potentially hampered by the low representation of infected cells in the total population, a situation that becomes extreme in those cases, like that of *Tomato yellow leaf curl virus* (TYLCV), in which the virus is restricted to phloem companion cells.

**Results:**

In order to gain insight into how different the transcriptional landscapes of TYLCV-infected cells or whole tissues of TYLCV-infected plants might be, here we compare the transcriptional changes in leaf patches infected with TYLCV by agroinfiltration or in systemic leaves of TYLCV-infected plants in *Nicotiana benthamiana*. Our results show that, in agreement with previous works, infection by TYLCV induces a dramatic transcriptional reprogramming; the detected changes, however, are not equivalent in local and systemic infections, with a much larger number of genes differentially expressed locally, and some genes responding in an opposite manner. Interestingly, a transcriptional repression of the auxin signalling pathway and a transcriptional activation of the ethylene signalling pathway were detected in both local and systemically infected samples. A transcriptional activation of defence was also detectable in both cases. Comparison with the transcriptional changes induced by systemic infection by the geminivirus *Tobacco curly shoot virus* (TbSV) shows common subsets of up- and down-regulated genes similarly affected by both viral species, unveiling a common transcriptional repression of terpenoid biosynthesis, a process also suppressed by the geminivirus *Tomato yellow leaf curl China virus.*

**Conclusions:**

Taken together, the results presented here not only offer insight into the transcriptional changes derived from the infection by TYLCV in *N. benthamiana*, but also demonstrate that the resolution provided by local and systemic infection approaches largely differs, highlighting the urge to come up with a better system to gain an accurate view of the molecular and physiological changes caused by the viral invasion.

**Electronic supplementary material:**

The online version of this article (10.1186/s12864-019-5842-7) contains supplementary material, which is available to authorized users.

## Background

As intracellular parasites, viruses have evolved to create a cellular environment permissive for viral replication in susceptible hosts; for this purpose, viruses induce a re-wiring of the host’s physiology and development concomitant to the establishment of a successful infection. In plants, these virus-induced changes can be easily visualized and quantified, and infection by different viruses frequently produces some of a common array of symptoms, including stunting, chlorosis, and leaf curling. Possibly both enabling and resulting from these virus-triggered changes, infected hosts undergo a dramatic transcriptional reprogramming; the analysis of the modifications in the transcriptional landscape of the host upon the viral infection can shed light on the molecular processes underlying the outcome of virus-host interactions. Such transcriptional studies have proliferated in the past decade, possibly owing to technical advances allowing for in-depth sequencing, availability of genomic information, and the increased affordability of these approaches.

Geminiviruses are insect-transmitted DNA viruses causing severe diseases in crops worldwide, and currently pose a serious threat to food security; however, our understanding of the molecular basis of the infection is still partial, which limits the development of effective anti-geminiviral strategies for crop protection. The transcriptional changes triggered by the infection by geminiviruses have been studied in a number of plant-virus interactions [1–8]. When comparing the results obtained in these studies, few commonalities arise: plant hormone signaling pathways, especially those for jasmonates (JA) and brassinosteroids (BR), frequently appear as altered, although the direction of the change is not consistent [4, 5, 8, 9]; and cell cycle-related genes, which need to be reactivated in the virus-infected terminally differentiated cells to allow for viral DNA replication, are detected as differentially expressed in a couple of cases only [2, 9]. In an attempt to unveil the molecular basis of tolerance or recovery, Chen et al. (2013) and Gongora-Castillo et al. (2012) [3, 4] compared the transcriptome of susceptible or tolerant tomato cultivars infected with *Tomato yellow leaf curl virus* (TYLCV) and that of recovered and symptomatic leaves of pepper infected with *Pepper golden mosaic virus* (PepGMV), respectively, by RNA-seq; however, and somewhat surprisingly, only limited differences were detected in both cases.

One factor frequently neglected in these transcriptional studies of geminivirus-infected plants is the low representation of infected cells in the total population: in an average infection, only some cells will be supporting viral replication at a given time. This situation becomes extreme in those cases, like that of TYLCV, in which the virus is restricted to phloem companion cells. The global transcriptome of infected plants will be the average of that in all cells, infected and non-infected, and all tissues: this not only creates a serious dilution issue, but also averages the potential transcriptional responses in infected and systemic, uninfected cells, which could be opposite, hence generating potentially misleading results difficult to interpret. However, and since to date no transcriptional profile of isolated infected cells is available, the extent to which this could be different to that obtained from complete aerial organs of an infected plant is unclear.

In order to gain insight into how different the transcriptional landscapes of TYLCV-infected cells or whole tissues of TYLCV-infected plants might be, here we compare the transcriptional changes in leaf patches infected with TYLCV by agroinfiltration or in systemic leaves of TYLCV-infected plants in *Nicotiana benthamiana* by RNA-seq. Strikingly, our results show that, as expected and in agreement with previous works, infection by TYLCV induces a dramatic transcriptional reprogramming; the detected changes, however, are not equivalent in local and systemic infections, with a much larger number of genes differentially expressed locally, and some genes responding in an opposite manner in local and systemic samples. Interestingly, a transcriptional repression of the auxin signaling pathway and a transcriptional activation of ethylene signaling and defence responses were detected both in local and systemic infections. Despite more limited changes detected in the systemically infected samples, comparison with the transcriptional changes induced by systemic infection by the geminivirus *Tobacco curly shoot virus* (TbSV) [5] unveiled common subsets of up- and down-regulated genes similarly affected by both viral species. Among the common biological processes potentially affected by the transcriptional changes we find terpenoid biosynthesis as transcriptionally repressed; notably, another geminivirus species, *Tomato yellow leaf curl China virus* (TYLCCN), has been shown to suppress terpenoid biosynthesis [10], making it tempting to speculate that depletion of terpenoids might be a requirement for geminiviruses to establish a successful infection in nature. Taken together, the results presented here not only shed light on the transcriptional changes derived from the infection by TYLCV in *N. benthamiana*, but also demonstrate that the resolution provided by local and systemic infection approaches largely differs, highlighting the urge to come up with a better system to gain an accurate view of the molecular and physiological changes caused by the viral invasion.

## Results

### Transcriptional changes upon local infection by TYLCV in *N. benthamiana*

From the observation that only a fraction of cells support active replication by a geminivirus at a given time [11, 12] logically follows the idea that an accurate study of the cellular changes triggered by the viral invasion will require the isolation of the infected cells specifically, and their comparison with similar cells from an uninfected sample. Analysis of whole organs of systemically infected plants, which is the common practice due to the lack of a more precise approach, would presumably result in potential dilution and masking issues. In order to test this idea, we decided to compare the transcriptional changes detectable by RNA sequencing (RNA-seq) upon local or systemic infection in the model plant *N. benthamiana* by the geminivirus TYLCV.

**Fig. 1:**
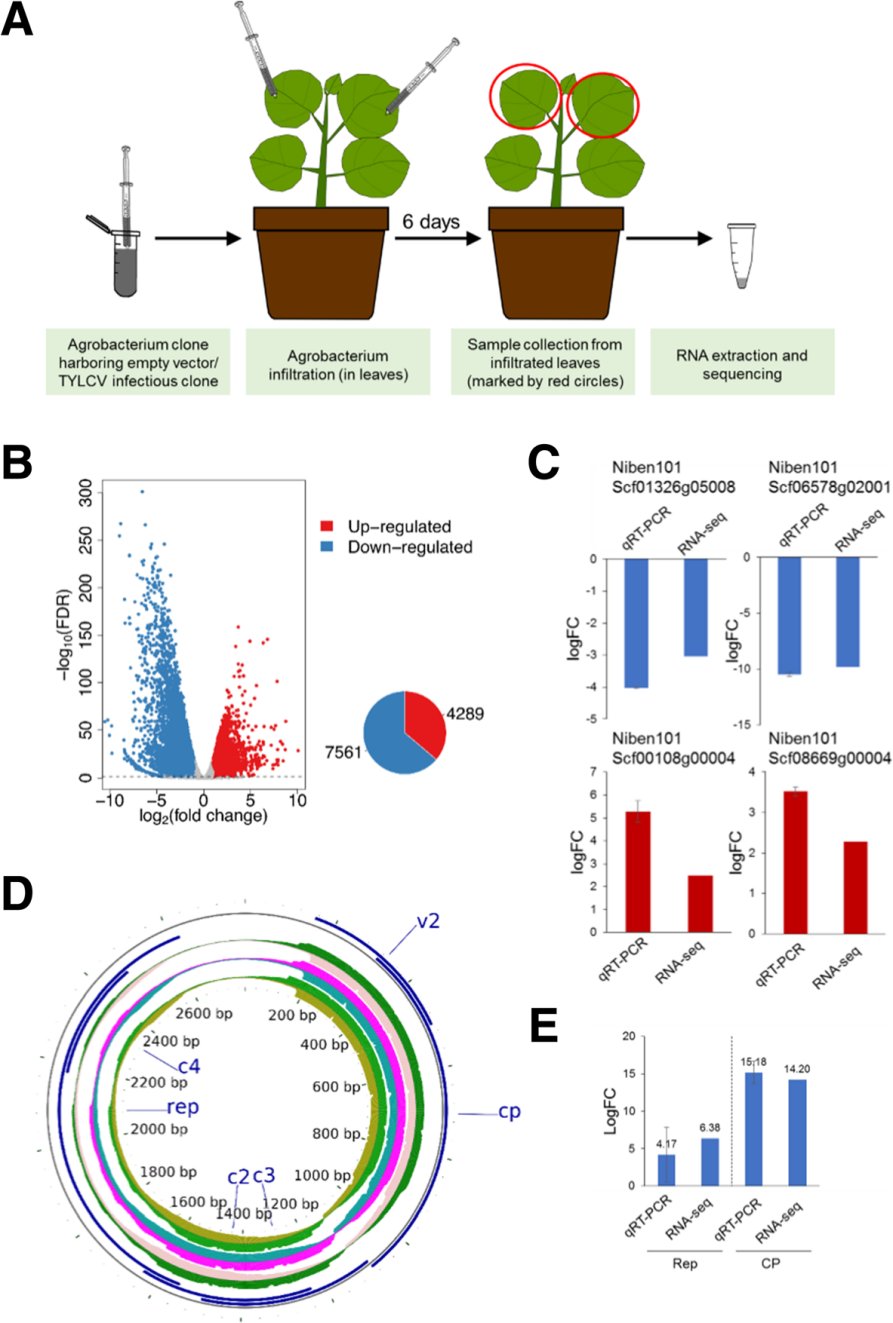
Transcriptional changes upon local infection by TYLCV in *N. benthamiana*. **a** Schematic representation of the experimental design. **b** Differentially expressed genes in the local infection are shown by volcano plot. The x-axis shows the log2 transformed gene expression fold change between infected and control samples. The y-axis indicates the negative log10 transformed adjusted *p*-values (FDR) of the differential expression test calculated by R package *edgeR*. The up-regulated and down-regulated genes are represented by red and blue dots, respectively. Pie chart shows the number of up−/down-regulated genes. **c** Validation of selected DEGs by qPCR. Values are the average of three biological replicates, relative to mock. *NbACT* was used as the normalizer. **d** Mapping of viral reads to the TYLCV genome. Three biological replicates (inner concentric circles) are represented; the upper side of each circle represents the virion (+) strand; the lower side of each circle represents the complementary (−) strand. ORFs are depicted in blue. Please note that the accumulated reads in the area containing the CP and V2 ORFs have been trimmed; the numbers of total reads are shown in Additional file 7: Table S2. **e** Validation of the expression of Rep and CP by qPCR. Expression values are relative to *NbACT*

The leaf patch infection results in the viral replication in most cells [11], which the virus must effectively manipulate to generate a permissive environment, hence serving as a good surrogate system to study the viral infection. For the local infection, we performed agroinfection in leaf patches of four-week-old *N. benthamiana* leaves and took samples at 6 days post-infiltration (dpi) (Fig. 1a), when the virus is still actively replicating. In order to exclude the potential effect of the bacteria on plant transcription, *N. benthamiana* leaves inoculated with an Agrobacterium clone containing the empty vector were used as control; three independent biological replicates were used in each case. RNA-seq was performed by Illumina sequencing as indicated in the methods section. The raw HiSeq reads were filtered and trimmed, and between 34 and > 51 million clean pair-end reads were obtained per sample; these clean reads were mapped to the *N. benthamiana* draft genome (v1.0.1) from the Sol Genomics Network (ftp://ftp.solgenomics.net/genomes/Nicotiana_benthamiana/assemblies/)) with a mapping rate between 89 and > 98% (Additional file 6 and Table 1). The PCA analysis of the three biological replicates for TYLCV and control samples is shown in Additional file 1: Figure S1.

**Table 1:** Summary of the RNA-seq results

Infection	Sample	Replicate	Raw reads	Clean reads	Mapped reads	Mapping rate
local	EV	1	56,095,920	49,607,102	48,784,974	98.34%
local	EV	2	40,783,494	35,461,778	34,878,028	98.35%
local	EV	3	59,215,650	50,797,194	49,957,654	98.35%
local	TLCV	1	46,414,226	42,273,896	38,070,811	90.06%
local	TYLCV	2	40,583,678	34,799,420	31,263,478	89.84%
local	TYLCV	3	42,441,872	36,642,412	32,897,690	89.78%
systemic	EV	1	45,831,882	42,069,696	41,242,974	98.03%
systemic	EV	2	40,432,870	36,748,216	35,955,596	97.84%
systemic	EV	3	48,885,308	45,262,860	44,405,978	98.11%
systemic	TYLCV	1	47,349,296	43,508,220	42,484,166	97.65%
systemic	TYLCV	2	55,797,434	51,761,828	50,618,532	97.79%
systemic	TYLCV	3	49,035,824	45,797,334	44,821,767	97.87%

A total of 7561 and 4289 down- and up-regulated genes, respectively, were identified in these locally infected samples (Fig. 1b; Additional file 6: Table S1). The RNA-seq results were validated by qPCR analysis of selected genes (Fig. 1c).

The clean reads were also mapped to the TYLCV genome, with an average of 88,550 reads per million (RPM) (Fig. 1d; Additional file 7: Table S2). Unexpectedly, reads for both strands of the virus were detected throughout the viral genome with uneven and non-perfectly symmetrical distribution, and not restricted to the described open reading frames (ORFs); the number of reads was much higher in the region of the genome containing the V2 and CP (late) genes (Additional file 7: Table S2). The accumulation of viral reads was confirmed by qPCR to detect expression of the viral genes encoding the Rep (Replication-associated protein) and the CP (capsid protein), contained in the complementary and the virion strand of the viral genome, respectively (Fig. 1e).

### Transcriptional changes upon systemic infection by TYLCV in *N. benthamiana*

**Fig. 2:**
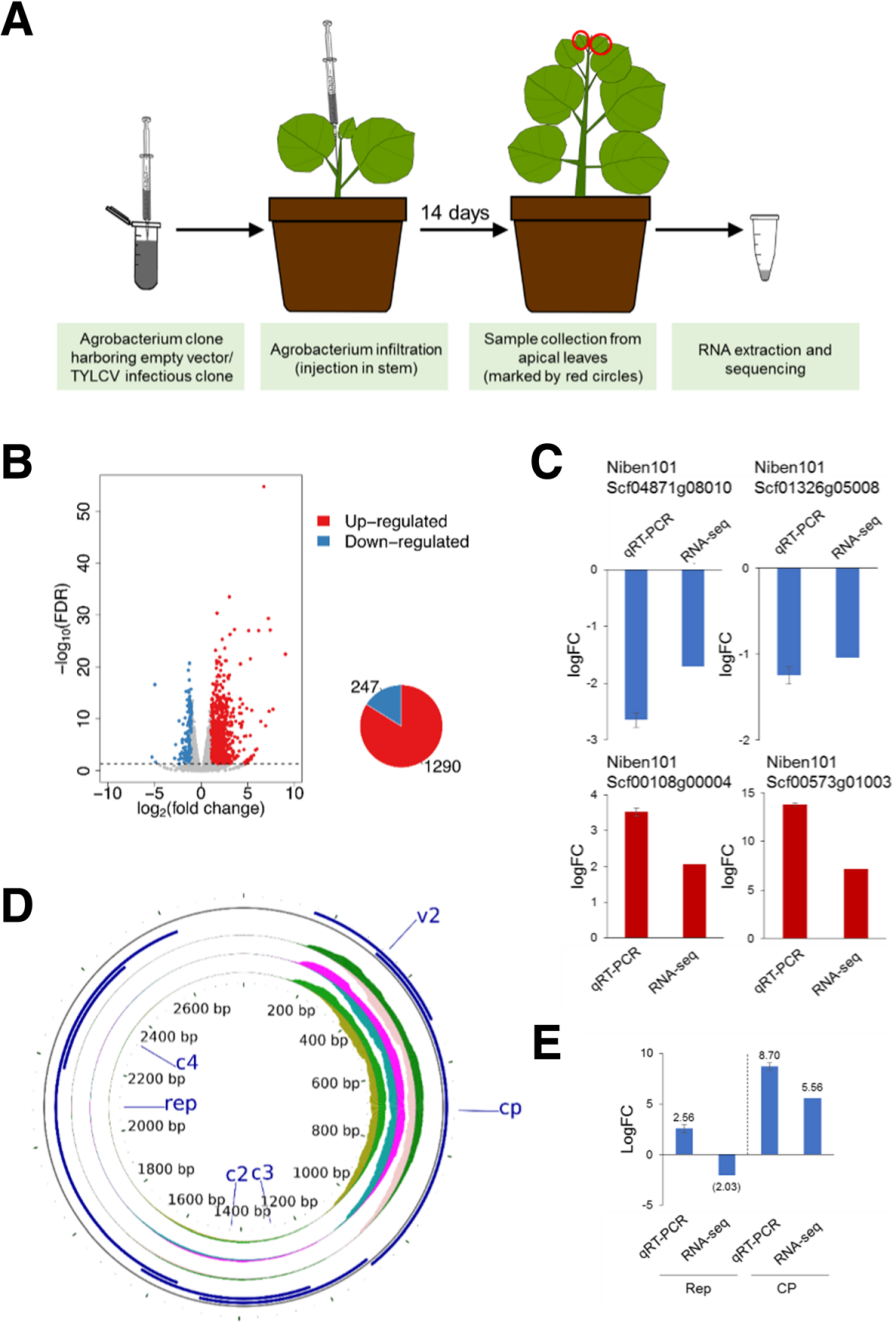
Transcriptional changes upon local infection by TYLCV in *N. benthamiana***. a** Schematic representation of the experimental design. **b** Differentially expressed genes in the systemic infection are shown by volcano plot. The x-axis shows the log2 transformed gene expression fold change between infected and control samples. The y-axis indicates the negative log10 transformed adjusted *p*-values (FDR) of the differential expression test calculated by R package *edgeR*. The up-regulated and down-regulated genes are represented by red and blue dots, respectively. Pie chart shows the number of up−/down-regulated genes. **c** Validation of selected DEGs by qPCR. Values are the average of three biological replicates, relative to mock. *NbACT* was used as the normalizer. **d** Mapping of viral reads to the TYLCV genome. Three biological replicates (inner concentric circles) are represented; the upper side of each circle represents the virion (+) strand; the lower side of each circle represents the complementary (−) strand. ORFs are depicted in blue. The number of total reads are shown in Additional file 7: Table S2. **e** Validation of the expression of Rep and CP by qPCR. Expression values are relative to *NbACT*

In order to analyze the transcriptional changes detectable during the systemic infection by TYLCV, we performed agroinfection of two-week-old *N. benthamiana* plants as described in [12], and took samples at 14 days post-infection (dpi) (Fig. 2a), when the virus is actively replicating in the apical leaves and the first symptoms have already appeared (Additional file 2: Figure S2). Apical leaves of *N. benthamiana* plants inoculated with an Agrobacterium clone containing the empty vector were used as control; three independent biological replicates were used in each case. RNA-seq was performed by Illumina sequencing as indicated in the methods section. The raw HiSeq reads were filtered and trimmed, and between 36 and > 52 million clean pair-end reads were obtained per sample; these clean reads were mapped to the *N. benthamiana* draft genome (v1.0.1) from the Sol Genomics Network (ftp://ftp.solgenomics.net/genomes/Nicotiana_benthamiana/assemblies/) with a mapping rate between 97 and > 98% (Additional file 6: Table S1). The PCA analysis of the three biological replicates for TYLCV and control samples is shown in Additional file 1: Figure S1.

A total of 247 and 1290 down- and up-regulated genes, respectively, were identified in these systemically infected samples (Fig. 2b; Additional file 8: Table S3). The RNA-seq results were validated by qPCR analysis of selected genes (Fig. 2c).

The clean reads were also mapped to the TYLCV genome, with an average of 3344 reads per million (RPM) (Fig. 2d; Additional file 7: Table S2). Also in this case, reads for both strands of the virus were detected throughout the viral genome with uneven and non-perfectly symmetrical distribution, and not restricted to the described ORFs; as observed in the locally infected samples, the number of reads was notably higher in the region of the genome containing the V2 and CP (late) genes (Fig. 2d; Additional file 7: Table S2). The accumulation of viral reads was confirmed by qPCR to detect expression of the viral genes encoding the Rep and the CP (Fig. 2e).

### Distinct landscapes of transcriptional changes detectable in local and systemic infections by TYLCV in *N. benthamiana*

In both the locally and the systemically infected samples used, the virus is actively replicating, and therefore those putative transcriptional changes underlying successful viral multiplication must have been established. The vast difference in the number of differentially expressed genes (DEGs) between these infected samples raises the possibility that the larger proportion of infected cells in the agroinfected leaf patches might provide an increase in resolution, which may result from a lower dilution of infection-induced transcriptional changes due to a higher infected-to-uninfected cell ratio and/or negligible masking from potential non-cell-autonomous plant responses to the viral infection.

**Fig. 3:**
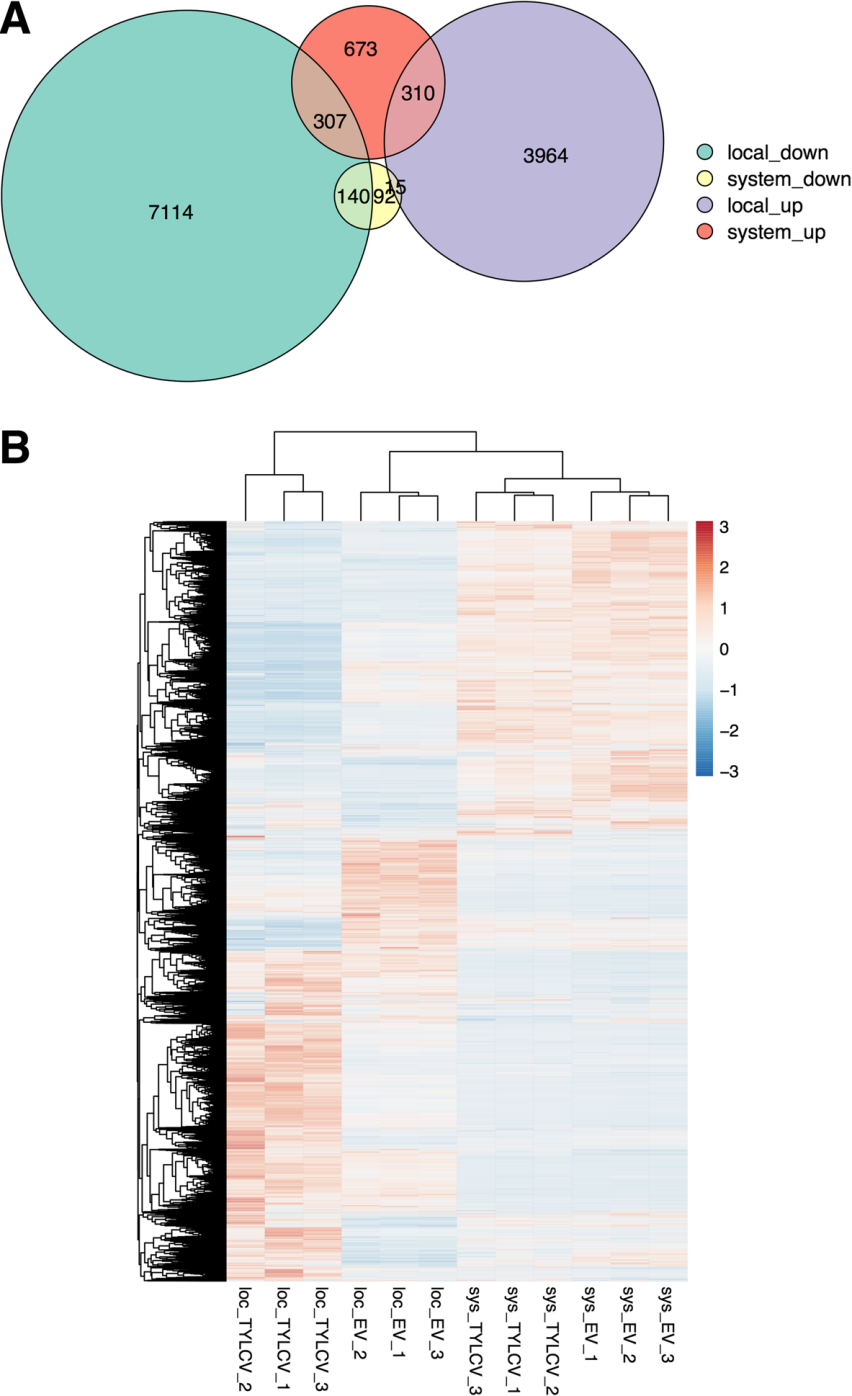
Different transcriptional changes are detected upon local and systemic infections by TYLCV in *N. benthamiana***. a** Venn diagram of DEGs in local and systemic infections. **b** The differential gene expressions of local and systemic infection samples are shown in heatmap with hierarchical clustering, which is drawn by R package *pheatmap*. The values in the gene expression matrix are centered and scaled in the row direction. The color bar indicates the Z-score. Loc: local infections; sys: systemic infections. EV: empty vector

In order to gain further insight into the differences between locally and systemically infected samples, we set out to compare the subsets of induced or repressed genes in each case. Strikingly, as shown in Fig. 3a, both the up- and the down-regulated genes in systemic infections show only a partial overlap with those in local infections: only 56.7% of the repressed genes are also repressed in local infections, while, surprisingly, 6% are induced; among the induced genes in systemic infections, only 24% are also up-regulated in local infections, with a 23.8% down-regulated in these samples. Hierarchical clustering (Fig. 3b) shows that the systemically infected samples cluster closer to their control than to the locally infected samples. Taken together, these results clearly indicate that the differences detected between datasets go beyond a higher sensitivity in locally infected samples, which would explain the quantitative differences in DEGs, but not the apparent opposite behavior of some of them.

**Fig. 4:**
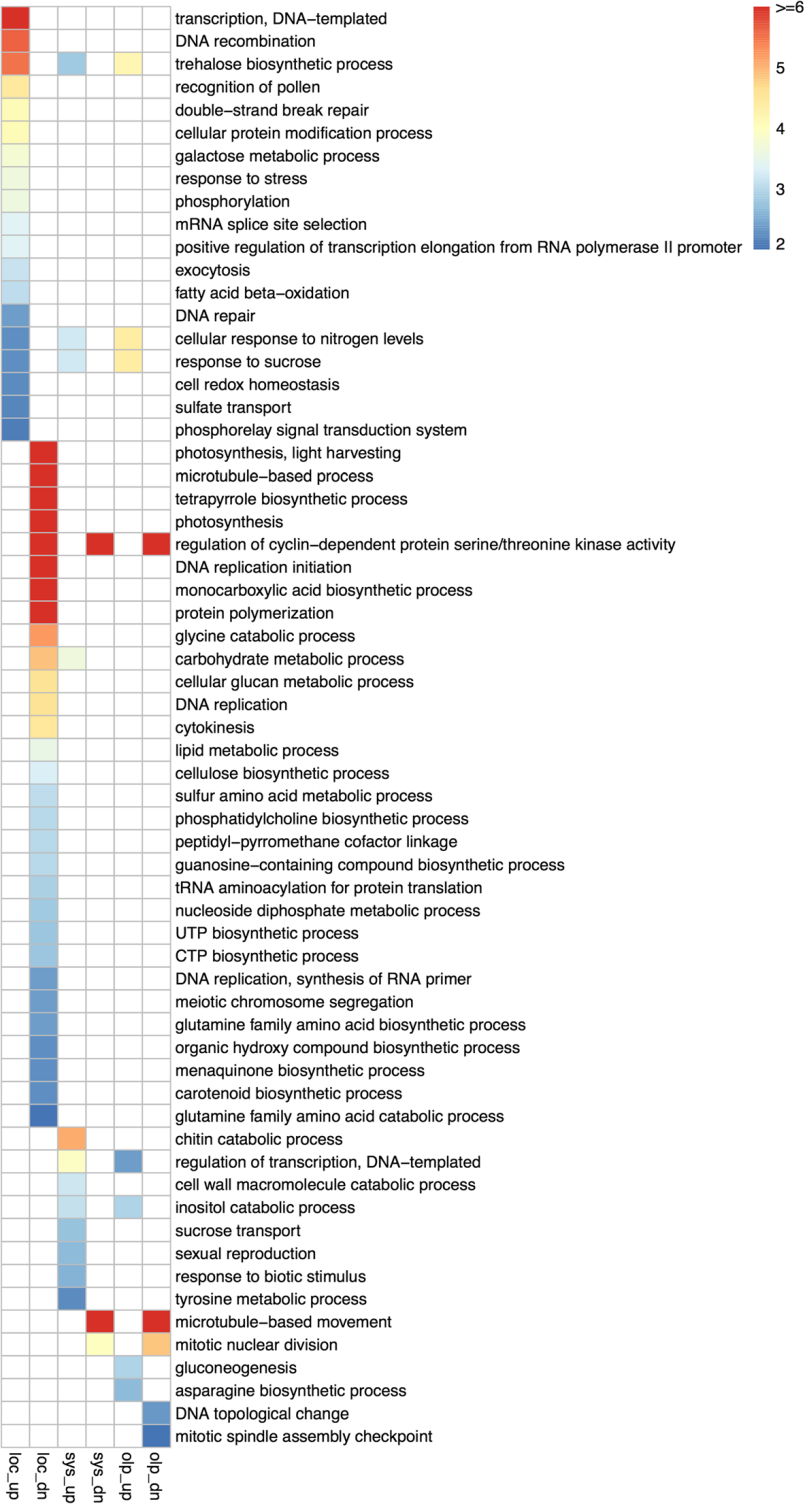
Functional enrichment analysis of differentially expressed genes in local and systemic TYLCV infections. Heatmap shows the Gene Ontology terms of biological processes that are significantly enriched (*p* < 0.01) in the up−/down-regulated genes in the local infection, the systemic infection, and their overlaps. Each square is colored according to the value of –log_10_(*p*), where *p* is the *p*-value for the significance of GO term enrichment. The color bar indicates the –log_10_(*p*). loc: local infection; sys: systemic infection; olp: overlapping between local and systemic infections. up: up-regulated; dn: down-regulated

**Fig. 5:**
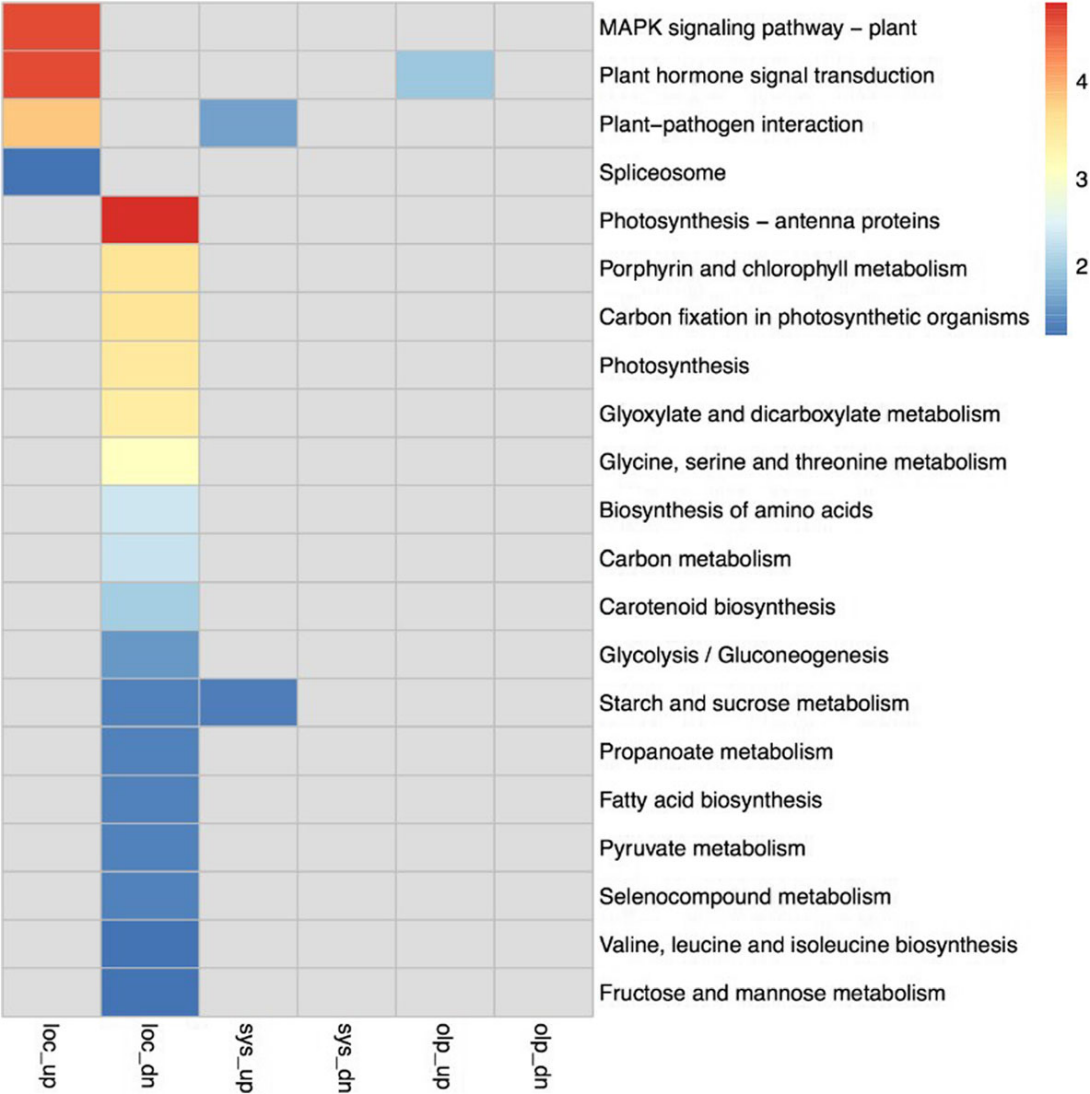
KEGG pathways of differentially expressed genes in local and systemic TYLCV infections. Heatmap shows the KEGG pathways that are significantly enriched (*p* < 0.05) in the up−/down-regulated genes in the local infection, the systemic infection, and their overlaps. Each square is colored according to the value of –log_10_(*p*), where *p* is the *p*-value for the significance of KEGG pathway enrichment. The color bar indicates the –log_10_(*p*). up: up-regulated; dn: down-regulated

With the aim of determining whether the distinct transcriptional landscapes of local and systemic infections may nevertheless result in similar functional outputs, we performed functional enrichment analysis using Gene Ontology and KEGG pathways annotations. As shown in Figs. 4 and 5 and Table 2, the overlap in over-represented GO categories (Biological Process Ontology) or KEGG pathways for systemic and local infections is only marginal. Common over-represented functional categories in both subsets of DEGs include trehalose biosynthetic process and defence response among the induced genes, and mitotic nuclear division and cellulose biosynthetic process among the repressed genes (Table 2).

**Table 2:** Significantly enriched GO terms (Biological Process Ontology) in the subsets of differentially expressed genes (*p*-value < 0.05)

GO.ID	Term	Annotated	Significant	Expected	*p*-value
Overrepresented in the subset of up-regulated in local infection					
GO:0006351	transcription, DNA-templated	2046	271	151.48	1.8E-22
GO:0006310	DNA recombination	47	15	3.48	2.5E-06
GO:0005992	trehalose biosynthetic process	34	12	2.52	3.1E-06
GO:0048544	recognition of pollen	69	16	5.11	0.000034
GO:0006302	double-strand break repair	16	7	1.18	0.000076
GO:0006464	cellular protein modification process	2490	237	184.35	0.00008
GO:0006012	galactose metabolic process	23	8	1.7	0.00016
GO:0006950	response to stress	873	108	64.63	0.00022
GO:0016310	phosphorylation	2148	196	159.03	0.00023
GO:0006376	mRNA splice site selection	6	4	0.44	0.0004
GO:0032968	positive regulation of transcription elongation from RNA polymerase II promoter	3	3	0.22	0.00041
GO:0006887	exocytosis	72	14	5.33	0.00071
GO:0006635	fatty acid beta-oxidation	7	4	0.52	0.00087
GO:0006281	DNA repair	271	38	20.06	0.00415
GO:0043562	cellular response to nitrogen levels	2	2	0.15	0.00548
GO:0009744	response to sucrose	2	2	0.15	0.00548
GO:0045454	cell redox homeostasis	229	28	16.95	0.00608
GO:0008272	sulfate transport	31	7	2.3	0.00653
GO:0000160	phosphorelay signal transduction system	102	15	7.55	0.00799
GO:0006334	nucleosome assembly	59	10	4.37	0.01074
GO:0009298	GDP-mannose biosynthetic process	7	3	0.52	0.0113
GO:0070966	nuclear-transcribed mRNA catabolic process, no-go decay	3	2	0.22	0.01562
GO:0070481	nuclear-transcribed mRNA catabolic process, non-stop decay	3	2	0.22	0.01562
GO:0071025	RNA surveillance	3	2	0.22	0.01562
GO:0015706	nitrate transport	3	2	0.22	0.01562
GO:0010167	response to nitrate	3	2	0.22	0.01562
GO:0007064	mitotic sister chromatid cohesion	8	3	0.59	0.01709
GO:0006032	chitin catabolic process	31	6	2.3	0.02419
GO:0035434	copper ion transmembrane transport	9	3	0.67	0.02425
GO:0050832	defense response to fungus	4	2	0.3	0.02972
GO:0042742	defense response to bacterium	4	2	0.3	0.02972
GO:0007050	cell cycle arrest	10	3	0.74	0.03277
GO:0006529	asparagine biosynthetic process	11	3	0.81	0.04263
GO:0006020	inositol metabolic process	11	3	0.81	0.04263
Overrepresented in the subset of down-regulated in local infection					
GO:0009765	photosynthesis, light harvesting	74	61	12.92	1E-30
GO:0007017	microtubule-based process	254	105	44.35	4.6E-19
GO:0033014	tetrapyrrole biosynthetic process	53	32	9.25	3.3E-12
GO:0015979	photosynthesis	387	169	67.57	4.9E-12
GO:0000079	regulation of cyclin-dependent protein serine/threonine kinase activity	48	29	8.38	3.4E-11
GO:0006270	DNA replication initiation	18	16	3.14	7.7E-11
GO:0072330	monocarboxylic acid biosynthetic process	171	61	29.85	8.1E-09
GO:0051258	protein polymerization	86	34	15.01	8.8E-09
GO:0006546	glycine catabolic process	11	9	1.92	5.8E-06
GO:0005975	carbohydrate metabolic process	1345	320	234.82	0.000013
GO:0006073	cellular glucan metabolic process	142	56	24.79	0.000026
GO:0006260	DNA replication	119	55	20.78	0.000027
GO:0000910	cytokinesis	24	12	4.19	0.000033
GO:0006629	lipid metabolic process	928	234	162.02	0.00028
GO:0030244	cellulose biosynthetic process	62	22	10.82	0.00051
GO:0000096	sulfur amino acid metabolic process	50	18	8.73	0.00084
GO:0006656	phosphatidylcholine biosynthetic process	4	4	0.7	0.00093
GO:0018160	peptidyl-pyrromethane cofactor linkage	4	4	0.7	0.00093
GO:1901070	guanosine-containing compound biosynthetic process	17	9	2.97	0.00094
GO:0006418	tRNA aminoacylation for protein translation	95	29	16.59	0.00125
GO:0009132	nucleoside diphosphate metabolic process	135	40	23.57	0.00155
GO:0006228	UTP biosynthetic process	15	8	2.62	0.00171
GO:0006241	CTP biosynthetic process	15	8	2.62	0.00171
GO:0006269	DNA replication, synthesis of RNA primer	5	4	0.87	0.00399
GO:0045132	meiotic chromosome segregation	5	4	0.87	0.00399
GO:0009084	glutamine family amino acid biosynthetic process	31	12	5.41	0.00416
GO:1901617	organic hydroxy compound biosynthetic process	23	14	4.02	0.00531
GO:0009234	menaquinone biosynthetic process	3	3	0.52	0.00532
GO:0016117	carotenoid biosynthetic process	14	7	2.44	0.00536
GO:0009065	glutamine family amino acid catabolic process	12	6	2.1	0.00997
GO:0046168	glycerol-3-phosphate catabolic process	6	4	1.05	0.01031
GO:0015671	oxygen transport	6	4	1.05	0.01031
GO:0006801	superoxide metabolic process	23	9	4.02	0.01161
GO:0007076	mitotic chromosome condensation	13	6	2.27	0.01582
GO:0006166	purine ribonucleoside salvage	4	3	0.7	0.01849
GO:0006096	glycolytic process	114	29	19.9	0.02014
GO:0046274	lignin catabolic process	33	11	5.76	0.02045
GO:0042549	photosystem II stabilization	7	4	1.22	0.02075
GO:0042026	protein refolding	14	6	2.44	0.02366
GO:0019307	mannose biosynthetic process	2	2	0.35	0.03047
GO:0007155	cell adhesion	2	2	0.35	0.03047
GO:0045038	protein import into chloroplast thylakoid membrane	2	2	0.35	0.03047
GO:0048478	replication fork protection	2	2	0.35	0.03047
GO:0007623	circadian rhythm	2	2	0.35	0.03047
GO:0010027	thylakoid membrane organization	2	2	0.35	0.03047
GO:0006450	regulation of translational fidelity	2	2	0.35	0.03047
GO:0030259	lipid glycosylation	15	6	2.62	0.03373
GO:0009082	branched-chain amino acid biosynthetic process	23	8	4.02	0.03537
GO:0006168	adenine salvage	5	3	0.87	0.04023
GO:0010207	photosystem II assembly	5	3	0.87	0.04023
GO:0006353	DNA-templated transcription, termination	5	3	0.87	0.04023
GO:0006662	glycerol ether metabolic process	68	18	11.87	0.04094
Overrepresented in the subset of up-regulated in systemic infection					
GO:0006032	chitin catabolic process	31	7	0.78	9.1E-06
GO:0006355	regulation of transcription, DNA-templated	1764	69	44.11	0.00012
GO:0005975	carbohydrate metabolic process	1345	63	33.64	0.00022
GO:0043562	cellular response to nitrogen levels	2	2	0.05	0.00062
GO:0009744	response to sucrose	2	2	0.05	0.00062
GO:0016998	cell wall macromolecule catabolic process	29	5	0.73	0.00069
GO:0019310	inositol catabolic process	8	3	0.2	0.00079
GO:0005992	trehalose biosynthetic process	34	5	0.85	0.00146
GO:0015770	sucrose transport	3	2	0.08	0.00184
GO:0019953	sexual reproduction	11	3	0.28	0.00221
GO:0009607	response to biotic stimulus	43	6	1.08	0.00271
GO:0006570	tyrosine metabolic process	5	2	0.13	0.00594
GO:0006094	gluconeogenesis	8	2	0.2	0.01581
GO:0006952	defense response	112	8	2.8	0.01862
GO:0006541	glutamine metabolic process	23	3	0.58	0.01898
GO:0000272	polysaccharide catabolic process	25	3	0.63	0.02376
GO:0006662	glycerol ether metabolic process	68	5	1.7	0.02756
GO:0003333	amino acid transmembrane transport	46	4	1.15	0.02758
GO:0006529	asparagine biosynthetic process	11	2	0.28	0.02956
GO:0008283	cell proliferation	11	2	0.28	0.02956
GO:0044264	cellular polysaccharide metabolic process	148	7	3.7	0.03961
GO:0008272	sulfate transport	31	3	0.78	0.04163
GO:0006857	oligopeptide transport	33	3	0.83	0.04874
Overrepresented in the subset of down-regulated in systemic infection					
GO:0007018	microtubule-based movement	129	11	0.7	1.2E-10
GO:0000079	regulation of cyclin-dependent protein serine/threonine kinase activity	48	7	0.26	7.2E-09
GO:0007067	mitotic nuclear division	51	5	0.28	0.0001
GO:0016114	terpenoid biosynthetic process	30	2	0.16	0.0116
GO:0006265	DNA topological change	30	2	0.16	0.0116
GO:0007094	mitotic spindle assembly checkpoint	3	1	0.02	0.0163
GO:0006952	defense response	112	3	0.61	0.0235
GO:0009813	flavonoid biosynthetic process	6	1	0.03	0.0323
GO:0030244	cellulose biosynthetic process	62	2	0.34	0.0452
Overrepresented in the subset of up-regulated genes common to local and systemic infections					
GO:0043562	cellular response to nitrogen levels	2	2	0.01	0.000041
GO:0009744	response to sucrose	2	2	0.01	0.000041
GO:0005992	trehalose biosynthetic process	34	4	0.22	0.000066
GO:0006094	gluconeogenesis	8	2	0.05	0.0011
GO:0019310	inositol catabolic process	8	2	0.05	0.0011
GO:0006529	asparagine biosynthetic process	11	2	0.07	0.0022
GO:0006355	regulation of transcription, DNA-templated	1764	21	11.37	0.0043
GO:0007205	protein kinase C-activating G-protein coupled receptor signaling pathway	24	2	0.15	0.0104
GO:0006659	phosphatidylserine biosynthetic process	3	1	0.02	0.0192
GO:0042742	defense response to bacterium	4	1	0.03	0.0255
GO:0006562	proline catabolic process	4	1	0.03	0.0255
GO:0050832	defense response to fungus	4	1	0.03	0.0255
GO:0006570	tyrosine metabolic process	5	1	0.03	0.0318
GO:0003333	amino acid transmembrane transport	46	2	0.3	0.0355
GO:0009064	glutamine family amino acid metabolic process	57	3	0.37	0.0453
Overrepresented in the subset of down-regulated genes common to local and systemic infections					
GO:0007018	microtubule-based movement	129	10	0.43	1.4E-11
GO:0000079	regulation of cyclin-dependent protein serine/threonine kinase activity	48	7	0.16	2.1E-10
GO:0007067	mitotic nuclear division	51	5	0.17	0.000014
GO:0006265	DNA topological change	30	2	0.1	0.0045
GO:0007094	mitotic spindle assembly checkpoint	3	1	0.01	0.0099
GO:0030244	cellulose biosynthetic process	62	2	0.21	0.0181
GO:0006606	protein import into nucleus	12	1	0.04	0.0392

Three KEGG pathways from Fig. 5 were selected to be graphically displayed, allowing for visualization and easy comparison of the transcriptional regulation of their components: MAPK signaling pathway (Additional file 3: Figure S3), Plant hormone signal transduction (Additional file 4: Figure S4), and Plant-pathogen interaction (Additional file 5: Figure S5). As observed in Additional file 3: Fig. S3 and Additional file 4: Figure S4, although not statistically significant in all cases, both local and systemic infections have an impact on these pathways, with the local infections having the strongest effect. As previously mentioned, the presence of the virus seems to activate plant defence responses (Additional file 3: Figures S3; Additional file 5: Figure S5). Notably, both types of infection trigger a detectable transcriptional repression of auxin signaling and a transcriptional activation of ethylene signaling, while only local infections resulted in a repression of the brassinosteroid signaling pathway (Additional file 4: Figure S4).

### TYLCV and TbSCV modify the expression of a set of common genes upon systemic infection in *N. benthamiana*

**Fig. 6:**
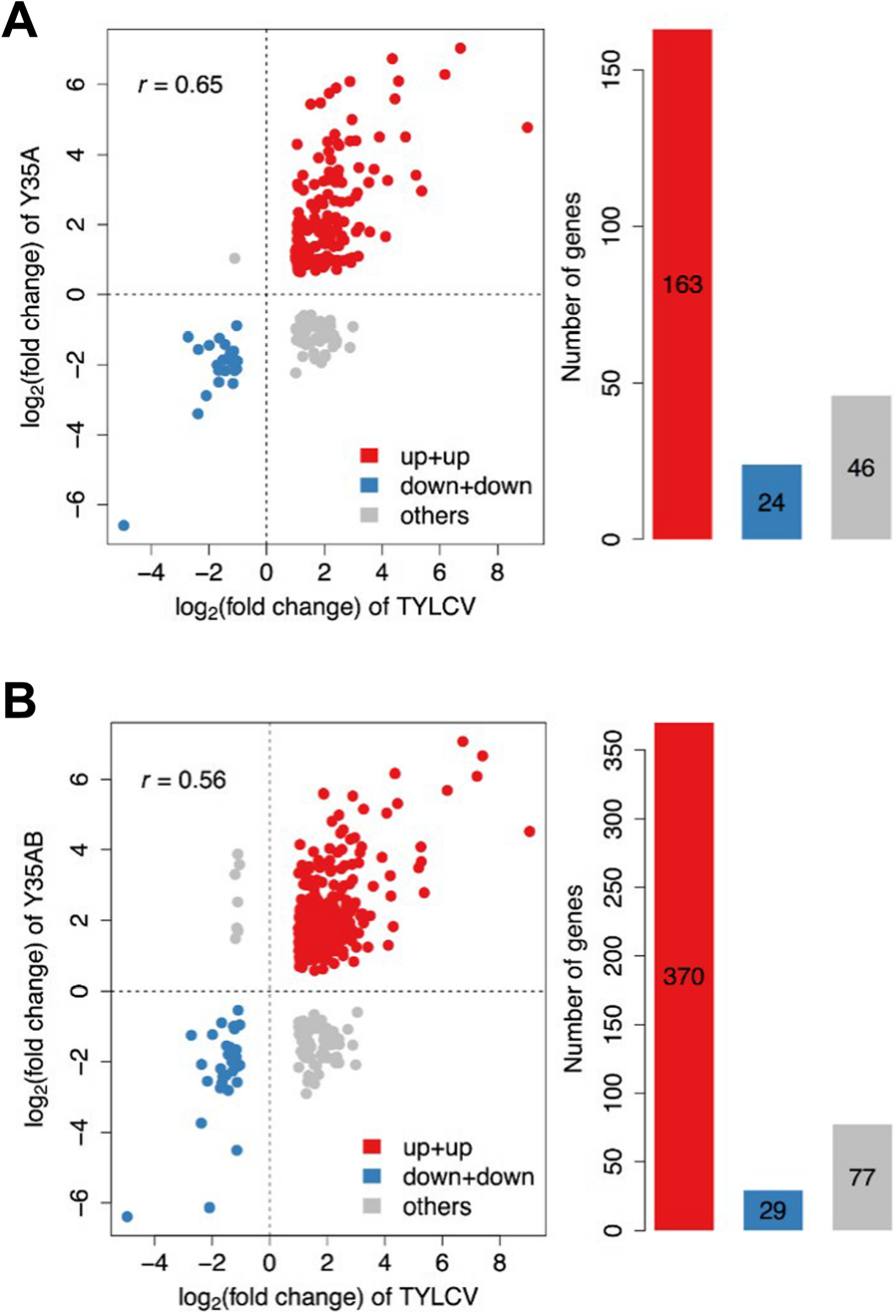
Systemic infection by TYLCV and TbSCV in *N. benthamiana* trigger partially overlapping transcriptional changes**.** The transcriptional changes triggered by the systemic infection by TYLCV were compared to those triggered by the geminivirus TbSCV, without (**a**) or with (**b**) its associated satellite, in *N. benthamiana*; the TbSCV data are from Li et al., 2018

In an attempt to identify potential central targets of the transcriptional geminiviral manipulation and/or effectors of the plant anti-geminiviral response, we decided to compare the transcriptional changes triggered by systemic infections by TYLCV and the geminivirus TbSCV, in combination with or without its associated satellite, in *N. benthamiana* [5]. Remarkably, a proportion of DEG were commonly affected by both TYLCV and TbSCV (12.6% of up-regulated genes by TYLCV infection, and 9.7% of down-regulated genes by TYLCV infection) (Fig. 6a); the proportion of induced, but not repressed, genes largely increased (to 28.7% of up-regulated genes by TYLCV infection) when TbSCV was inoculated in combination with its satellite (Fig. 6b), suggesting that some of the virulence functions provided by this ancillary molecule result in the activation of host genes and are already encoded in the TYLCV genome. Functional enrichment analysis of the genes commonly activated or repressed by TYLCV and TbSCV revealed the existence of a number of GO categories over-represented in these subsets (Table 3), including trehalose biosynthetic process and defence responses (in the common up-regulated gene set), and terpenoid biosynthetic process (in the common down-regulated gene set). Interestingly, a third geminiviral species, TYLCCN, has been proven to suppress terpenoid biosynthesis and release, and this effect in turn improves performance of its insect vector, the whitefly *Bemisia tabaci* [10]. Considering this negative regulation by three different geminivirus species, it is tempting to speculate that depletion of terpenoids is a requirement for geminiviruses to establish a successful infection in nature, perhaps at least partly through an indirect effect on favouring the insect vector-virus mutualism.

**Table 3:** GO enrichment of DEGs common to systemic infections by TYLCV and TbSCV in *N. benthamiana*. Over-represented GO categories (Biological Process Ontology) in the different subsets of DEGs represented in Fig. 6

GO.ID	Term	Annotated	Significant	Expected	*p* value
Y35A_up+up					
GO:0006108	malate metabolic process	36	3	0.13	0.00027
GO:0046470	phosphatidylcholine metabolic process	19	2	0.07	0.00197
GO:0000272	polysaccharide catabolic process	25	2	0.09	0.00341
GO:0016998	cell wall macromolecule catabolic process	29	2	0.1	0.00457
GO:0006032	chitin catabolic process	31	2	0.11	0.00521
GO:0045454	cell redox homeostasis	229	4	0.8	0.00835
GO:0050832	defense response to fungus	4	1	0.01	0.01386
GO:0042742	defense response to bacterium	4	1	0.01	0.01386
GO:0046373	L-arabinose metabolic process	5	1	0.02	0.0173
GO:1902358	sulfate transmembrane transport	7	1	0.02	0.02413
GO:0019953	sexual reproduction	11	1	0.04	0.03767
Y35A_down+down					
GO:0016114	terpenoid biosynthetic process	30	2	0.02	0.00028
GO:0009813	flavonoid biosynthetic process	6	1	0	0.00498
GO:0006012	galactose metabolic process	23	1	0.02	0.01897
Y35AB_up+up					
GO:0006355	regulation of transcription, DNA-templated	1764	27	11.74	0.000032
GO:0006529	asparagine biosynthetic process	11	2	0.07	0.0023
GO:0006952	defense response	112	4	0.75	0.0067
GO:0046470	phosphatidylcholine metabolic process	19	2	0.13	0.007
GO:0006571	tyrosine biosynthetic process	3	1	0.02	0.0198
GO:0005992	trehalose biosynthetic process	34	2	0.23	0.0215
GO:0005975	carbohydrate metabolic process	1345	19	8.95	0.0215
GO:0006979	response to oxidative stress	257	5	1.71	0.0288
GO:0006421	asparaginyl-tRNA aminoacylation	5	1	0.03	0.0328
GO:0046373	L-arabinose metabolic process	5	1	0.03	0.0328
GO:0009607	response to biotic stimulus	43	2	0.29	0.0332
Y35AB_down+down					
GO:0016114	terpenoid biosynthetic process	30	2	0.03	0.00032
GO:0009813	flavonoid biosynthetic process	6	1	0.01	0.00529

## Discussion

In this work, we describe and compare the genome-wide transcriptional changes detectable by RNA-seq occurring in *N. benthamiana* upon local or systemic infection by the geminivirus TYLCV. Our results show that, as expected and in agreement with previous works, infection by TYLCV causes a strong transcriptional reprogramming in the host; however, the detectable changes are more dramatic in our local infection system. It is possible that the local infection offers higher resolution owing to lower dilution of the infected cells; however, the opposite behavior of some DEGs in local and systemic samples suggests that the absence of transcriptional changes coming from non-infected cells and masking local changes also plays a role in the differences detected. Additionally, we cannot rule out that some differences between the two infected samples could be due to differences in the stage of the infection: although in both cases the virus is actively replicating in infected cells, in the leaf patch assay the infection is supposed to be more or less synchronized, while in the systemic infection a mixture of cells at different phases of the infection is most likely present in the samples. Another potentially relevant difference between local and systemic samples is the presence of *Agrobacterium* in the former; nevertheless, *Agrobacterium* is also present in the respective control. It is relevant to note that, if local and systemic samples show different results due to dilution and masking issues, these problems would potentially not only affect the study of transcriptional changes, but also the analysis of other molecular or physiological alterations resulting from the viral infection.

Functional enrichment analyses can help us gain insight into the biological processes underlying infection by TYLCV (Tables 2 and 3). In locally infected samples, over-represented GO categories included DNA recombination among the up-regulated genes, and cytokinesis among the down-regulated genes. These processes are expected to be connected to the viral manipulation of DNA replication and cell cycle, and might not be detectable in the systemically infected samples as a result of dilution. Photosynthesis also appears as transcriptionally down-regulated; photosynthetic shut-down seems to be a common outcome in viral infections ([8, 13–16], among others). A transcriptional negative regulation of BR signaling can also be detected in locally infected samples; these results are in agreement with a recent work by Seo et al. (2018), in which suppression of BR signaling was shown to underpin symptom development triggered by TYLCV in tomato.

Among those categories over-represented in both local and systemic infections, we can find cellulose biosynthetic process as transcriptionally repressed, suggesting that the viral infection might be affecting cell wall composition; changes in cell wall dynamics have been recently shown as triggered by *Potato virus Y* [17] and *Rice tungro spherical virus* [18], and in the latter case they have been proposed to correlate with virus-induced stunting. Intriguingly, both viruses negatively impact the cellulose biosynthetic machinery; whether impaired cellulose biosynthesis is a general plant response to the viral invasion is an idea that will require further investigation. Auxin signaling is also transcriptionally repressed upon the viral infection; these changes may also mediate or modulate the impact of the viral infection on plant development.

The identification of common subsets of up- and down-regulated genes in the systemic infections by TYLCV and TbSV [5] indicates that geminiviral manipulation of the cell and/or plant defence responses to geminiviral infection follow common transcriptional routes in different geminivirus/*N. benthamiana* interactions. Perhaps of particular interest is the finding that defence responses are activated in response to the viral infection; this observation reveals that these geminiviruses are being efficiently perceived as non-self by the plant, which in turn triggers a defence response. Although the activated plant defence reponses are not sufficient to fend off the virus, since the infection is established successfully, the fact that the plant is capable of detecting these pathogens in the first place paves the way for future engineering of the host, potentially boosting defences downstream of perception of the virus and hence tilting the balance in favour of the plant. Another pathway that emerges as a potential valuable target for engineering anti-geminiviral resistance is terpenoid biosynthesis, which is transcriptionally repressed by TYLCV and TbSV and has been proven to be suppressed by TYLCCN [10], raising the idea that its down-regulation might underpin a successful geminiviral infection.

Our results support the idea that the leaf patch assay entails great potential for the study of geminiviruses. Not only does this system result in a relatively synchronic infection and provides high resolution to detect virus-induced changes, but it also allows for the study of mutant viruses. All TYLCV null mutants for single genes, with the exception of those mutated in Rep, are capable of replicating their genome, but unable to infect the plant systemically; the use of local infections makes it possible to analyze the differences between the cellular changes triggered by wild-type and mutant viruses, therefore providing insight into the function of the viral genes in the context of the infection. An inherent limitation of this surrogate system, however, is the inevitable loss of cell type specificity: while TYLCV naturally infects phloem companion cells exclusively, in a leaf patch assay the virus is forced to replicate in mesophyll cells. Another obvious shortcoming is the impossibility of studying those mechanisms involved in cell-to-cell or long-distance transport, or the interactions between virus and vector.

All things considered, and while the analysis of systemic and local infections has provided and will continue to provide useful insight into the molecular events underlying the infection by geminiviruses, both approaches are imperfect for a number of reasons, as mentioned above. In order to considerably deepen our view, offering a substantial leap in our understanding of the molecular and physiological changes occurring during the plant-geminivirus interaction, isolating those infected cells, ideally based on the stage of the infection, will be crucial. Several approaches would enable the isolation of geminivirus infected cells: the use of transgenic plants harbouring a replicon-based system to label those cells sustaining active viral replication (like those described in [11]) could be combined with Fluorescence Activated Cell Sorting (FACS) or Laser Capture Microdissection (LCM), leading to the separation of infected from non-infected cells; high-throughput single-cell sequencing would also allow the unbiased identification and analysis of those cells containing the virus in the context of the infected plant. The increase in precision and resolution provided by the isolation of infected cells and their comparison to uninfected cells in the same plant will foreseeably result in an unprecedented view of the molecular landscaping triggered by the viral invasion.

## Conclusions

Our results show that TYLCV induces a dramatic transcriptional reprogramming in *N. benthamiana*, the detection of which largely differs in local and systemic infections. Nevertheless, some responses, including a transcriptional repression of the auxin signaling pathway and a transcriptional activation of defence, can be commonly detected.

Comparison with the transcriptional changes induced by systemic infection by the geminivirus TbSV shows common subsets of up- and down-regulated genes similarly affected by both viral species, among which the suppression of terpenoid biosynthesis might be a general change triggered by geminiviruses*.* Taken together, our results not only provide insight into the transcriptional changes resulting from the infection by TYLCV in *N. benthamiana*, but also highlight the need to come up with an optimized system to gain a precise overview of the molecular and physiological changes caused in the host by the viral invasion.

## Methods

### Plant material and growth conditions

Wild-type *N. benthamiana* plants were grown in a controlled growth chamber in long day conditions (16 h light/8 h dark) at 25 °C.

### Viral infections

The TYLCV infectious clone is described in [19, 20]; it contains a partial dimer of the TYLCV genome (AJ489258; [21]) in the pGWB501 vector [22]. *Agrobacterium tumefaciens* GV3101 strain was used for the delivery of TYLCV infectious clone and empty pGWB501 vector. *Agrobacterium* cells carrying these constructs were liquid-cultured in LB with appropriate antibiotics at 28 °C overnight. Bacterial cultures were centrifuged at 4000 g for 10 min and resuspended in the infiltration buffer (10 mM MgCl_2_, 10 mM MES pH 5.6, 150 μM acetosyringone) to an OD_600_ = 0.5. Bacterial suspensions were incubated in the buffer at room temperature and in the dark for 4 h before using them to infiltrate 4-week-old *N. benthamiana* for leave patch assays (local infections) and three-week-old *N. benthamiana* for systemic infection as described in [12].

### RNA extraction

Total RNA was extracted from 8 mm leaf discs using the RNeasy plant mini kit (Qiagen) following the manufacturer’s instructions.

### RNA sequencing

Transcriptome analyses were performed at the Genomic Core Facility, Shanghai Center for Plant Stress Biology, CAS. Three biological replicates were used. Total RNA (1 μg) from each sample was used for library preparation with NEBNext Ultra Directional RNA Library Prep Kit for Illumina (New England BioLabs, E7420L) following the manufacturer’s instructions. Prepared libraries were assessed for quality using NGS High-Sensitivity kit on a Fragment Analyzer (AATI) and for quantity using Qubit 2.0 fluorometer (Thermo Fisher Scientific). All libraries were sequenced in paired-end 125 bases protocol (PE125) on an Illumina HiSeq sequencer.

### Quantitative RT-PCR

First-strand cDNA synthesis was performed with the iScriptTM cDNA Synthesis Kit (Bio-Rad #1708890) according to the manufacturer’s instructions. For qPCR reactions, the reaction mixture consisted of cDNA first-strand template, primers (500 nM each) and iTaqTM Universal SYBRR Green Supermix (Bio-Rad, #1725120). qPCR was performed in a BioRad CFX96 real-time system. Expression result was determined using the comparative Ct method (2^-ΔΔCt^). Primers used are described in Additional file 9: Table S4. *NbACT* was used as the reference gene, using primers described in [23].

### Preprocessing of RNA-Seq data

We cleaned the paired-end reads by Trimimomatic [24] (version 0.36). After trimming the adapter sequence, removing low quality bases and filtering short reads, clear read pairs were retained for further analysis.

### Mapping and quantification of TYLCV reads

Cleaned reads were mapped to TYLCV DNA (GenBank: AJ489258.1) and its six ORFs by HISAT [25] (version 2.1.0) with default parameters. The RPM (Reads per Million) was used to quantify the expression level of each ORF and the whole viral genome. The read coverage of each base on the reference DNA was calculated by samtools [26] (version 1.5) with maximum coverage depth 8000 (−d 8000) and normalized to RPM. The expression level and read coverage were calculated for forward and reverse strand, respectively. The circular viral genome and read coverage of RNA-Seq data were visualized by CGView [27].

### Reads mapping and quantification of *N. benthamiana* genes

The *N. benthamiana* draft genome sequence [28] (v1.0.1) was downloaded from the Sol Genomics Network (ftp://ftp.solgenomics.net/genomes/Nicotiana_benthamiana/assemblies/). Cleaned reads were mapped to the genome sequence by HISAT with default parameters. Number of reads that were mapped to each *N. benthamiana* gene was calculated with the *htseq-count* script in HTSeq [28].

### Differential gene expression analysis

EdgeR [29] was used to identify genes that were differentially expressed between control and experiment samples. Genes with at least two-fold change in expression and had a FDR < 0.05 were considered differentially expressed genes (DEGs).

### GO enrichment analysis

The Gene Ontology (GO) terms assigned to *N. benthamiana* genes were extracted from annotation file downloaded from the SGN (ftp://ftp.solgenomics.net/genomes/Nicotiana_benthamiana/annotation/Niben101/). GO enrichment analysis of DEGs was implemented by topGO [30] with custom gene-to-GOs mapping annotations.

### KEGG pathway enrichment analysis

Since *N. benthamiana* is not supported by KEGG, we searched its ortholog genes in *Arabidopsis* by BLAST+ (version 2.5.0, evalue = 1e-5). The ortholog genes of the DEGs were used to perform KEGG enrichment analysis by clusterProfiler [31]. The enriched KEGG pathways were visualized with DEGs by Pathview [32].

## Additional files

Additional file 1: **Figure S1.** PCA analysis of the RNA-seq datasets (local and systemic TYLCV infections) (JPG 529 kb)

Additional file 2: **Figure S2.** Pictures of plants systemically infected by TYLCV at the time of sample collection (JPG 299 kb)

Additional file 3: **Figure S3.** Differentially expressed genes of local (A) and systemic (B) TYLCV infections in the MAPK signaling pathway. The log_2_ transformed expression fold changes of DEGs are converted to pseudo colors using default limit (e.g. from − 1 to 1), and mapped to KEGG pathway by R package *pathview*. The up- and down- regulated genes are labeled in red and green, respectively. (JPG 919 kb)

Additional file 4: **Figure S4.** Differentially expressed genes of local (A) and systemic (B) TYLCV infections in the plant hormone signal transduction pathway. The log_2_ transformed expression fold changes of DEGs are converted to pseudo colors using default limit (e.g. from − 1 to 1), and mapped to KEGG pathway by R package *pathview*. The up- and down- regulated genes are labeled in red and green, respectively. (JPG 759 kb)

Additional file 5: **Figure S5.** Differentially expressed genes of local (A) and systemic (B) TYLCV infections in the plant-pathogen interaction pathway. The log_2_ transformed expression fold changes of DEGs are converted to pseudo colors using default limit (e.g. from − 1 to 1), and mapped to KEGG pathway by R package *pathview*. The up- and down- regulated genes are labeled in red and green, respectively. (JPG 640 kb)

Additional file 6: **Table S1.** DEGs in local TYLCV infections (XLSX 1067 kb)

Additional file 7: **Table S2.** Reads mapping to the viral genome in local and systemic infections (XLSX 10 kb)

Additional file 8: **Table S3.** DEGs in systemic TYLCV infections (XLSX 146 kb)

Additional file 9: **Table S4.** Primers used in this work (DOCX 14 kb)

## Data Availability

All data generated or analysed during this study are included in this published article (and its supplementary information files).
