# Supplementary figures and images for: Transcriptional reprogramming caused by the geminivirus Tomato yellow leaf curl virus in local or systemic infections in Nicotiana benthamiana
Source: BMC Genomics. 2019 Jul 4;20:542. doi: 10.1186/s12864-019-5842-7 (PMC6611054; doi:10.1186/s12864-019-5842-7)

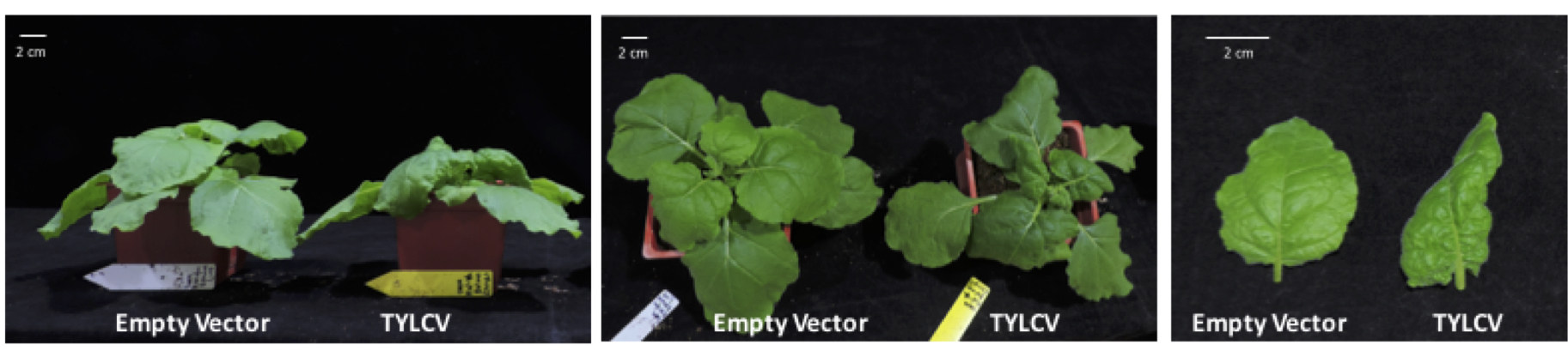

Supplement: Supplementary file 1 — Figure S1. PCA analysis of the RNA-seq datasets (local and systemic TYLCV infections) (JPG 529 kb) [file 12864_2019_5842_MOESM1_ESM.jpg]

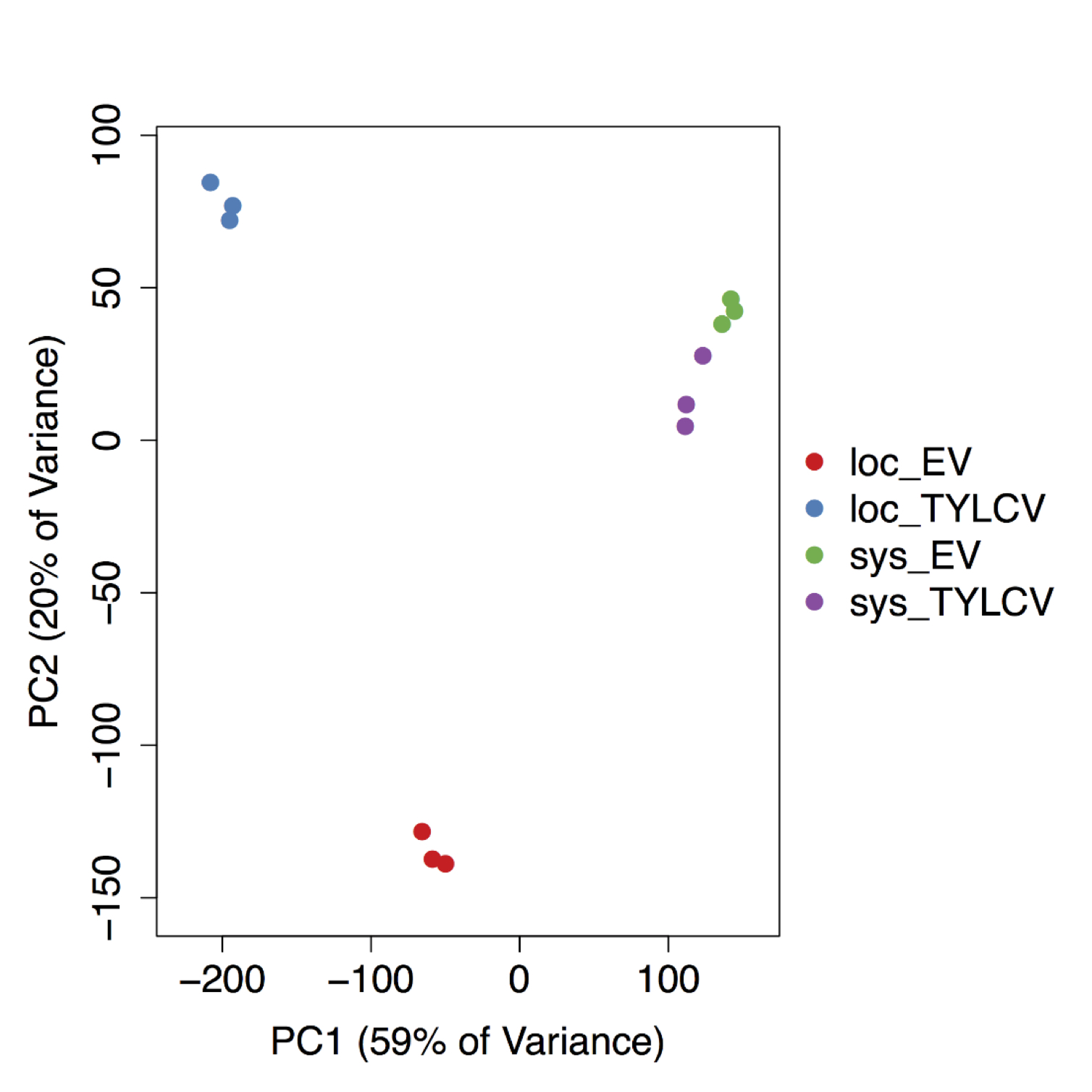

Supplement: Supplementary file 2 — Figure S2. Pictures of plants systemically infected by TYLCV at the time of sample collection (JPG 299 kb) [file 12864_2019_5842_MOESM2_ESM.jpg]

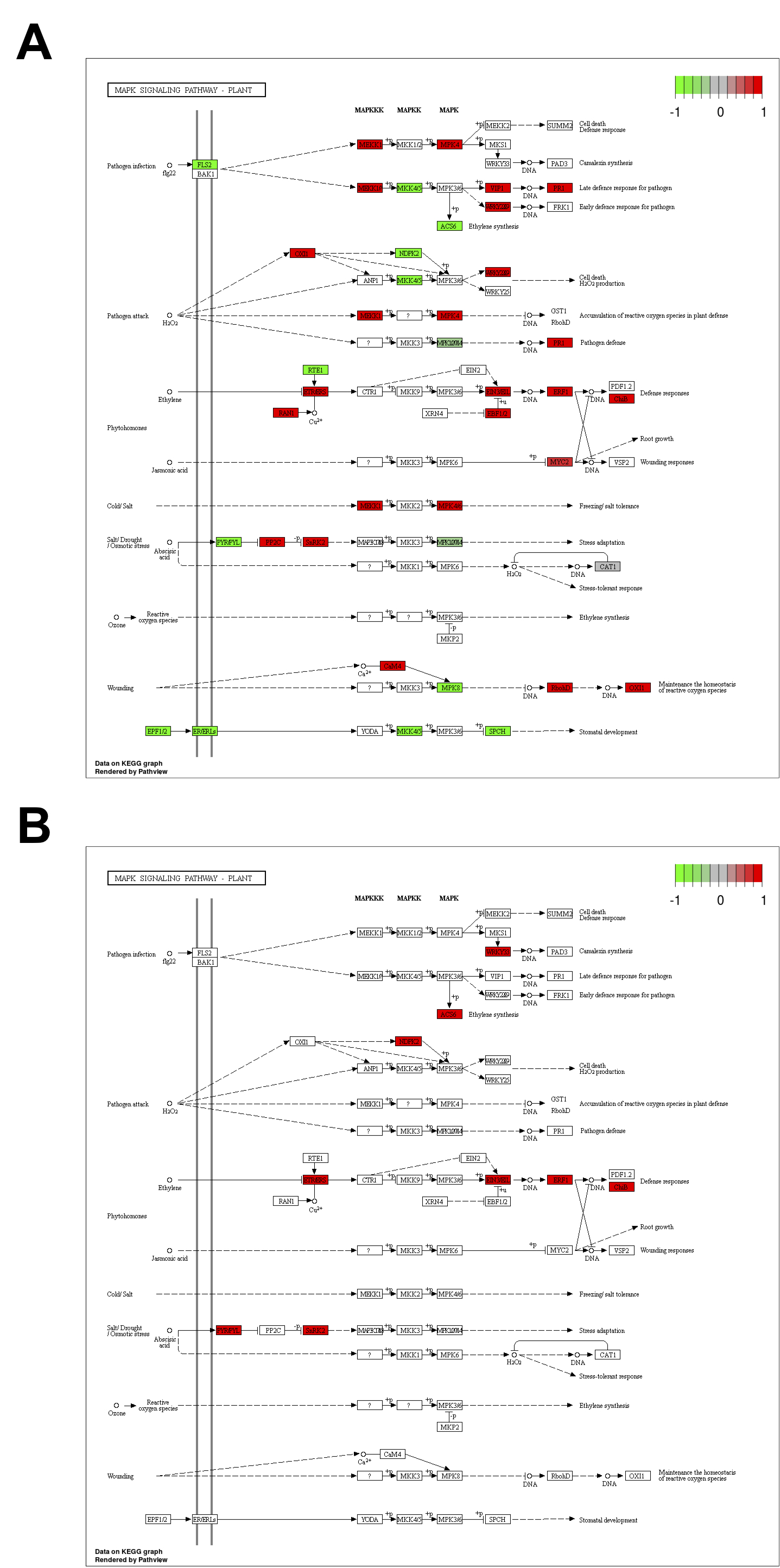

Supplement: Supplementary file 3 — Figure S3. Differentially expressed genes of local (A) and systemic (B) TYLCV infections in the MAPK signaling pathway. The log2 transformed expression fold changes of DEGs are converted to pseudo colors using default limit (e.g. from − 1 to 1), and mapped to KEGG pathway by R package pathview. The up- and down- regulated genes are labeled in red and green, respectively. (JPG 919 kb) [file 12864_2019_5842_MOESM3_ESM.jpg]

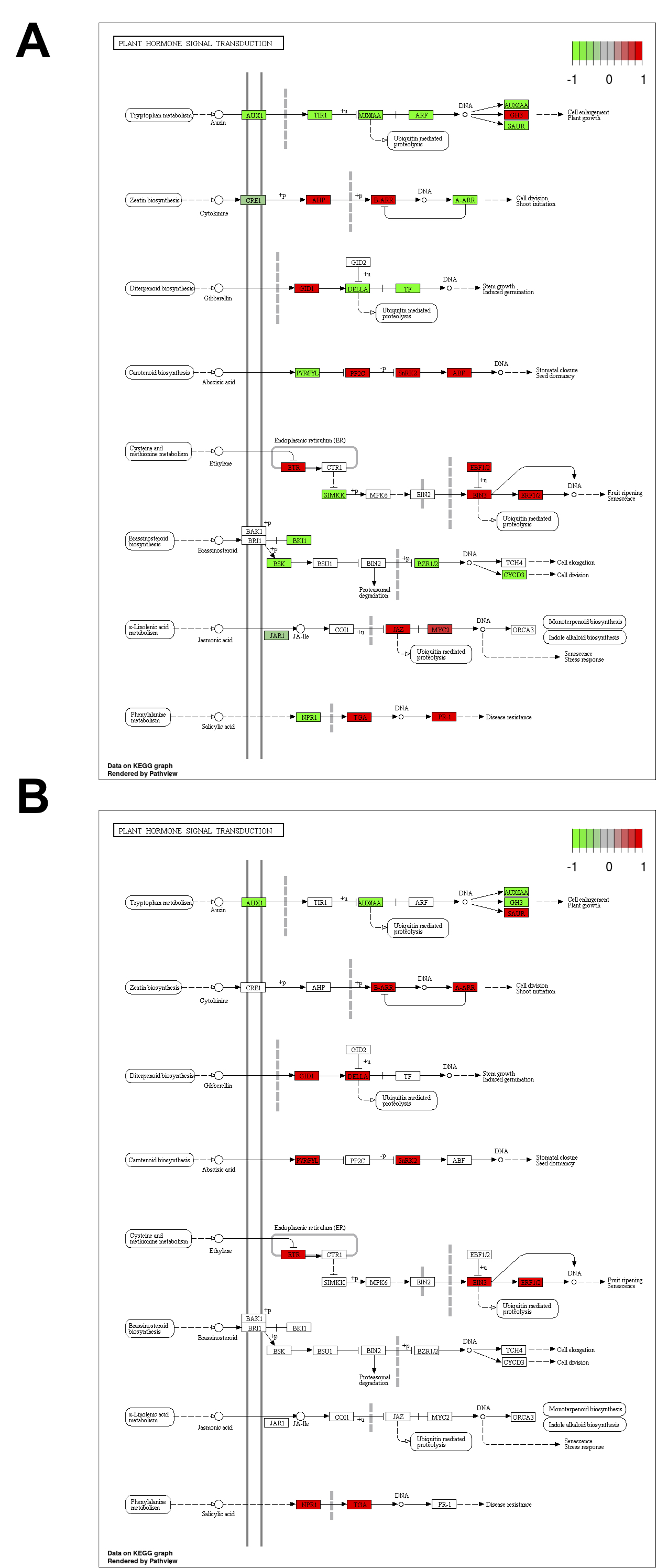

Supplement: Supplementary file 4 — Figure S4. Differentially expressed genes of local (A) and systemic (B) TYLCV infections in the plant hormone signal transduction pathway. The log2 transformed expression fold changes of DEGs are converted to pseudo colors using default limit (e.g. from − 1 to 1), and mapped to KEGG pathway by R package pathview. The up- and down- regulated genes are labeled in red and green, respectively. (JPG 759 kb) [file 12864_2019_5842_MOESM4_ESM.jpg]

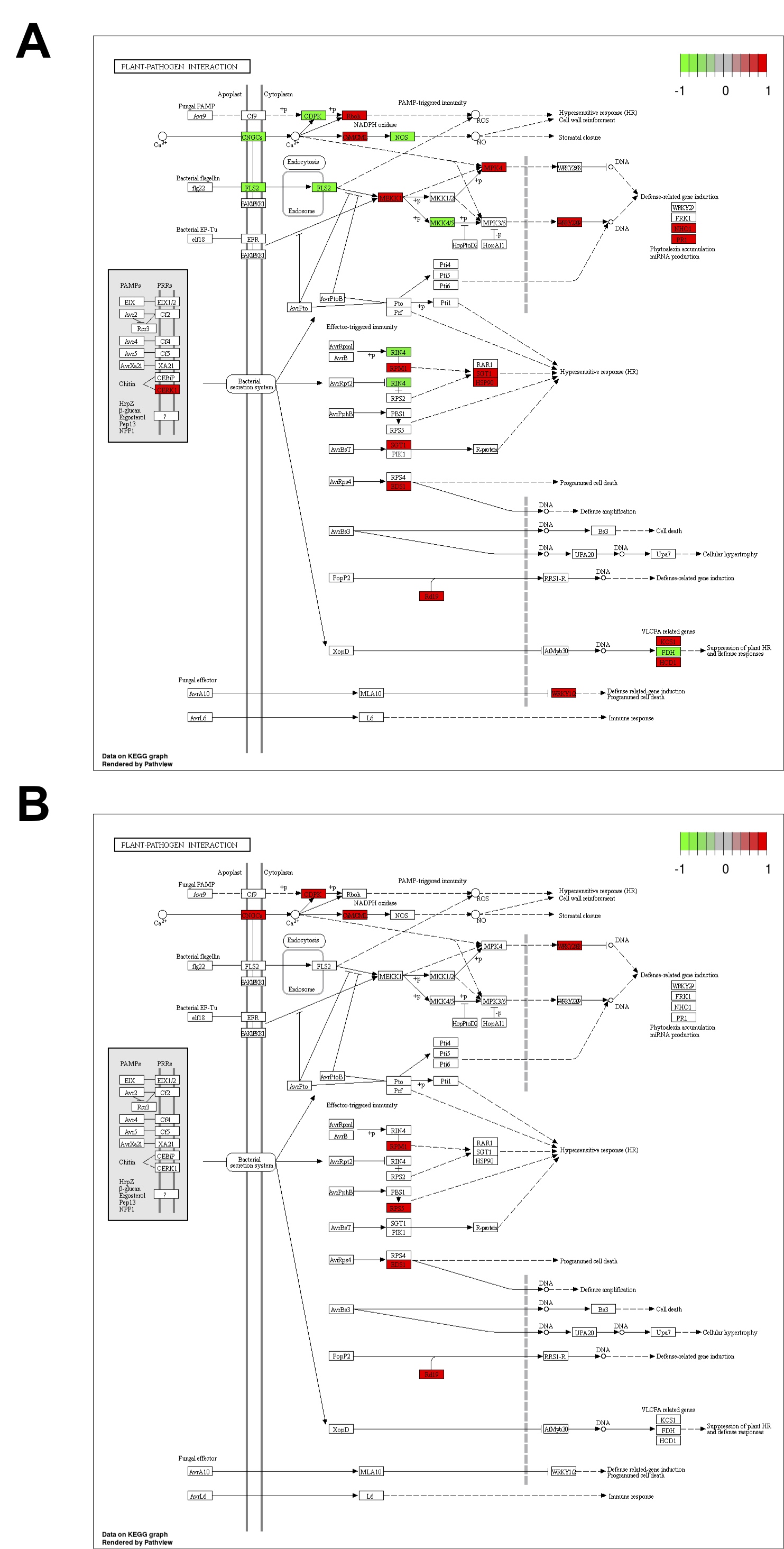

Supplement: Supplementary file 5 — Figure S5. Differentially expressed genes of local (A) and systemic (B) TYLCV infections in the plant-pathogen interaction pathway. The log2 transformed expression fold changes of DEGs are converted to pseudo colors using default limit (e.g. from − 1 to 1), and mapped to KEGG pathway by R package pathview. The up- and down- regulated genes are labeled in red and green, respectively. (JPG 640 kb) [file 12864_2019_5842_MOESM5_ESM.jpg]
