# Supplementary material for: Transcriptional reprogramming caused by the geminivirus Tomato yellow leaf curl virus in local or systemic infections in Nicotiana benthamiana
Source: BMC Genomics. 2019 Jul 4;20:542. doi: 10.1186/s12864-019-5842-7 (PMC6611054; doi:10.1186/s12864-019-5842-7)
Supplement: Supplementary file 9 — Table S4. Primers used in this work (DOCX 14 kb) [file 12864_2019_5842_MOESM9_ESM.docx]

| **Table S4 Primers used in this work.** | | | | |
| --- | --- | --- | --- | --- |
| **Gene** | **Primer sequence (5'->3')** | **Tm** | **Product length** | **Efficiency** |
| Niben101Scf08669g00004 | Fw: CGCCTTAATCCTCATTTCAACCG | 60.24 | 72 | 103.60% |
|  | Rv: ACCCGAGAAATTCCCACGAGAT | 61.48 |  |  |
| Niben101Scf00108g00004 | Fw: AAGGTTAGCACCCGAAGCTC | 60.04 | 127 | 98.10% |
|  | Rv: CCCATAATCCATTGGCTGAAAGC | 60.24 |  |  |
| Niben101Scf01326g05008 | Fw: CCTACAACTCCGTGCTTTCG | 58.93 | 104 | 99.80% |
|  | Rv: ATCCAGAGACTTTCGGCAAAT | 57.64 |  |  |
| Niben101Scf00573g01003 | Fw: CGTGTGCCGTCTATGGTAGAA | 59.87 | 76 | 94.00% |
|  | Rv: AGACTAAAACAGACTGCTGCT | 57.24 |  |  |
| Niben101Scf06578g02001 | Fw: AACTTACATCCCATGCCACCA | 59.64 | 90 | 86.20% |
|  | Rv: TGTGTCCTTTGGGCACTTCT | 59.45 |  |  |
| Niben101Scf04871g08010 | Fw: ACCGGCGGATGAAAGAACTC | 60.39 | 83 | 88.60% |
|  | Rv: CCGACCTTGGCTTTTGTTCG | 60.04 |  |  |
